# Is It Possible to Make Magnesia-Based Cement Environmentally Friendly?

**DOI:** 10.1021/acssuschemeng.4c05985

**Published:** 2024-11-06

**Authors:** Yongshan Tan, Shichang Liu, Mithila Achintha, Renjie Mi

**Affiliations:** † College of Civil Science and Engineering, Yangzhou University, Yangzhou 225127, China; ‡ School of Engineering, The University of Manchester, Manchester M13 9PL, United Kingdom; § Department of Engineering, University of Cambridge, Cambridge CB3 0FA, United Kingdom

**Keywords:** magnesia-based cement, life cycle assessment, environmental impacts, CO_2_ emissions

## Abstract

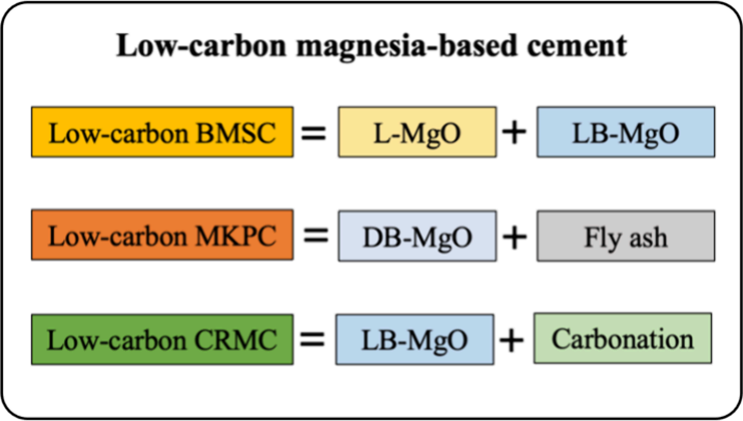

Magnesia-based cement is recognized for its outstanding mechanical properties, but its environmental impact has not been thoroughly evaluated. This paper employs a comprehensive life cycle assessment methodology to systematically analyze the environmental effects of four kinds of MgO and 10 kinds of magnesia-based cements based on the data in the literature. The impacts include CO_2_ emissions, fossil fuel depletion potential, and overall environmental impact indicators. The results indicate that using Salt Lake magnesium residue to prepare MgO, e.g., LB-MgO and DB-MgO, can reduce over 60% of CO_2_ emissions, compared with traditional MgO (e.g., L-MgO and D-MgO) prepared with magnesite. Utilizing supplementary cementitious materials (e.g., fly ash and ground granulated blast-furnace slag) as substitutes for clinker in basic sulfate magnesium cement (BMSC) and magnesium phosphate potassium cement (MKPC) can also reduce approximately 16 and 45% of carbon emissions, respectively. In addition, carbonation-reactive magnesium cement (CRMC), which involves carbonation curing and replacing traditional MgO with Salt Lake magnesium residue, is the most environmentally friendly magnesia-based cement with an overall environmental impact indicator of 0.00078.

## Abstract 2

Utilizing the Salt Lake magnesium residue and carbonation can produce environmentally friendly magnesia-based cement

## 1 Introduction

Magnesia-based cement prepared with magnesium oxide (MgO) is a promising alternative to calcium-based cement, such as ordinary Portland cement. Seawater and salt lakes worldwide provide abundant magnesium for the raw materials of magnesia-based cement,^1^ e.g., over 230 × 10^8^ tons of magnesium chloride in these salt lakes.^2,3^ In addition, magnesia-based cement performs better in some properties compared with ordinary Portland cement. For example, the density (e.g., 1600–1800 kg/m^3^) of basic magnesium sulfate cement is lower than that of ordinary Portland cement (e.g., 2200–2500 kg/m^3^).^4,5^ Moreover, the compressive strength of magnesium phosphate cement after curing 3 h was 36 MPa,^6,7^ which was higher than that (e.g., 20–30 MPa after curing 24 h^8,9^) of OPC. However, the carbon emissions of conventional magnesia-based cement are higher than those of ordinary Portland cement^10^ because manufacturing magnesium oxide generates more CO_2_ emissions compared to calcined limestone.^11,12^ Specifically, the decomposition of per ton magnesite resulted in 1.1 tons of CO_2_ emissions, while the figure for limestone calcination was 0.67 t/t.^13,14^

A series of strategies have been conducted to reduce the carbon footprint of magnesia-based cement. The first approach is using the byproduct containing magnesium oxide to substitute conventional magnesium oxide at different replacement rates. Tan et al.^15^ employed the calcination method to produce dead-burned boron-containing magnesium oxide (DB-MgO) with an optimal calcination temperature of 1000 °C, which was lower than that (i.e., 1400–2000 °C) of conventional dead-burned MgO. Such boron-containing magnesium oxide (B-MgO) was derived from boron-containing magnesium residues, which can be obtained during the extraction of lithium carbonate (Li_2_CO_3_) of the brine in Qarhan Salt Lake, China. It was observed that the magnesium phosphate potassium cement (MKPC) prepared with B-MgO achieved a compressive strength of 2.4 MPa after curing 3 h and increased to 58.1 MPa after curing 28 days. Furthermore, the final setting time of the MKPC was 15 min and 9 s. Similarly, Wu et al.^16^ prepared various light-burned magnesium oxides (L-MgOs) using the magnesium-enriched byproducts of the Salt Lake brine, and such MgOs were used to replace the L-MgO prepared from magnesite in basic magnesium sulfate cement (BMSC). The study revealed that the BMSC prepared using boron-containing L-MgO and the L-MgO prepared from magnesite had a 24 h compressive strength of up to 36.5 MPa and a 28-day compressive strength of up to 55.2 MPa, respectively.

The second approach involves utilizing industrial wastes to replace the clinker in magnesia-based cement. Zhang et al.^17^ studied the effects of different proportions of ground granulated blast-furnace slag (GGBS) and fly ash (FA) on the setting time and mechanical properties of BMSC. Their findings indicated that replacing 20% MgO by FA or GGBS resulted in final setting times of 199 or 173 min and 28 d compressive strengths of 70.7 or 69.2 MPa, respectively. Moreover, the inclusion of FA and GGBS significantly optimized the microstructure of BMSC, thereby enhancing its mechanical properties. Furthermore, Tan et al.^18,19^ reduced the production costs and the environmental impact of MKPC was achieved by adding FA and GGBS.

The third approach involves employing mineralization technology for magnesia-based cement, namely, carbonated reactive magnesia cement (CRMC). CRMC is prepared with light-burned MgO and has been extensively studied, owing to its carbon sequestration potential and exceptional mechanical properties. For example, Hay and Celik^20^ first moist cured CRMC samples at 22 ± 2 °C for 3 days, and then the specimens were carbonated in a sealed tank with a CO_2_ concentration of 20%, a relative humidity of 80%, and a temperature of 30 °C for 3 days. The strength of CRMC reached 41.9 MPa, which was 3 times higher than that of ordinary Portland cement at the same age. In addition, Liska and Al-Tabbaa^21^ discovered that up to 71% of CRMC can be carbonated, meaning that 1 kg of CRMC can sequester 0.78 kg of CO_2_. They also estimated that the carbon sequestration capacity of CRMC could be higher.

Life Cycle Assessment (LCA) has been used to evaluate the environmental impacts of some of the magnesia-based cement. For example, Miller and Myers^10^ employed LCA to quantify and compare the environmental effects of CRMC prepared with forsterite or magnesite. The CO_2_ emissions of CRMC produced from forsterite were 0.19 kg CO_2_-eq/kg cement, which were much lower than those of CRMC produced from magnesite. Meanwhile, the fossil fuel demand for CRMC produced from forsterite was 0.19 MJ/kg, which was lower than that for CRMC produced from magnesite. In addition, Ruan et al.^22^ explored the feasibility of utilizing waste brine from a desalination plant for CRMC production and examined its carbon emissions. They observed that the carbon footprint of waste brine production was 62% higher compared to that of CRMC production from magnesite. However, their study lacked an assessment of other environmental burdens linked to this specific production method. In addition, these studies only briefly compared the environmental effects of CRMC but did not offer a comprehensive and systematic quantification and assessment of the environmental effects of other magnesia-based cement.

Therefore, this paper studied the environmental effects of 10 kinds of magnesia-based cements using the LCA method. To assess the environmental effects of magnesia-based cement, a comprehensive assessment was conducted by using a wide range of environmental impact indicators. Based on the results of the Life Cycle Impact Assessment (LCIA), a series of recommendations were proposed. The primary objectives of this study are

(1) to compare the environmental effects of the entire production process of 10 types of magnesia-based cement utilizing the LCA methodology;
(2) to examine the environmental effects of two distinct sources of MgO: conventional MgO derived from the calcination of magnesite ore and MgO obtained by calcining magnesium residue, a byproduct of Qarhan Salt Lake; and
(3) to explore the environmental effects of magnesia-based cement with FA or GGBS.

This research has significantly enhanced the understanding of the environmental implications of different kinds of magnesia-based cements, offering robust data for the advancement of sustainable practices in magnesium cementitious material production.

## 2 Research Objectives and Methods

### 2.1 Research Objectives

#### 2.1.1 MgO

Cement clinker is a calcined and quenched material and is used as a reactive component in cement. The clinkers in this study encompassed four types of MgO: (1) L-MgO, (2) dead-burned magnesium oxide (D-MgO), (3) light-burned boron-containing magnesium oxide (LB-MgO), and (4) DB-MgO.

**Figure 1:**
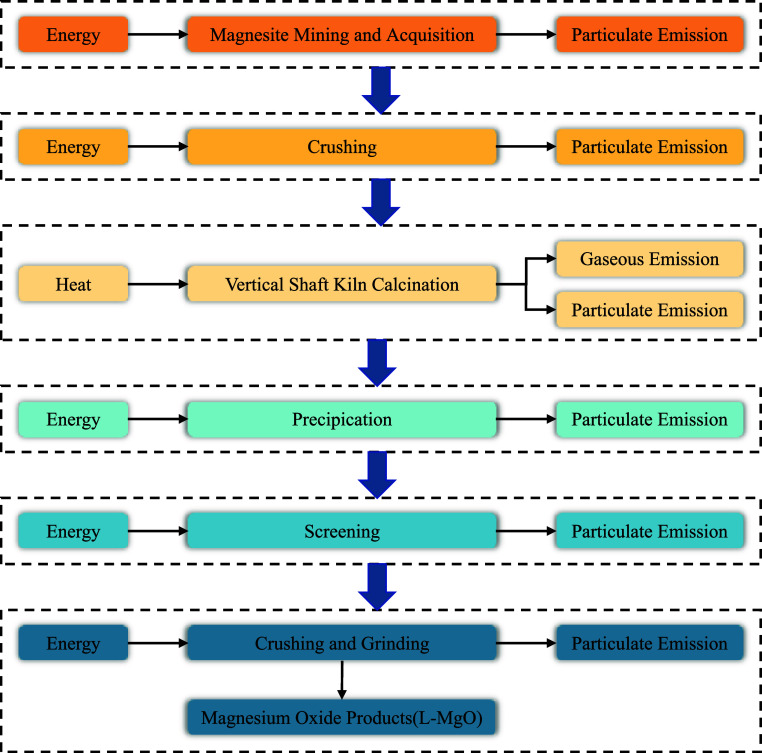
Energy consumption, heat consumption, and emissions during the production of L-MgO.

The production of L-MgO first involved the thermal treatment of magnesite at temperatures ranging from 750 to 850 °C. Subsequently, L-MgO was prepared using precipitation, flotation, and ball milling techniques.^23^Figure 1 elucidates the material consumption, energy and heat utilization, and emissions during the production of L-MgO. During the modeling phase, the inventory data for L-MgO were sourced from the study of Li et al.^24^ and are listed in Table S1 in the Supporting Information.

**Figure 2:**
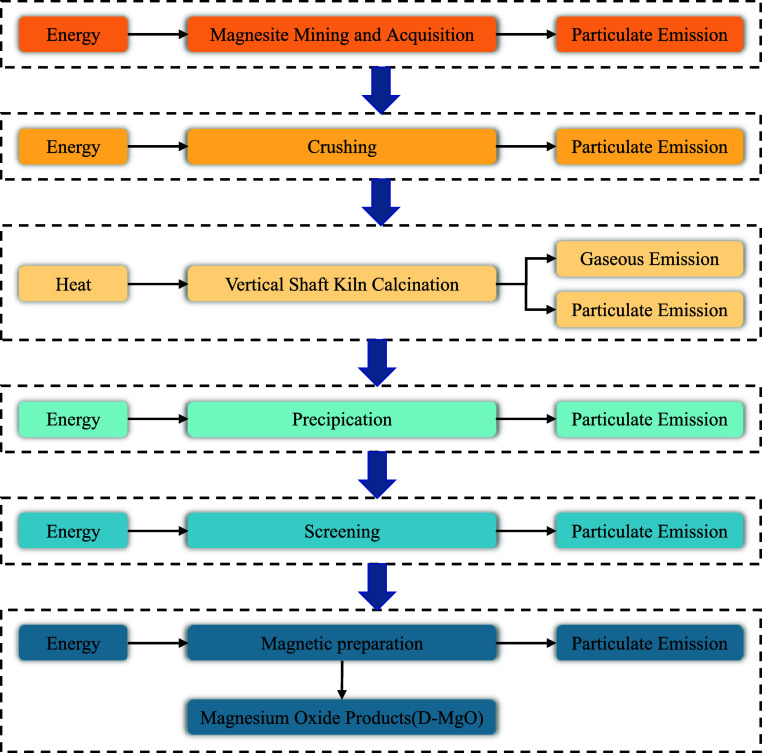
Energy consumption, heat consumption, and emissions in the production of D-MgO.

D-MgO was produced by magnesite at a temperature of 1500 °C, followed by ball milling. Given the absence of process inventory data for D-MgO in the literature, the production process of D-MgO was investigated in detail in this study to establish a detailed production process inventory.^23^ The calcination process was classified into one- and two-step methods. In the one-step approach, magnesite was crushed as a powder with specific granularity. Subsequently, it was placed into a vertical shaft kiln and directly calcined at temperatures ranging from 1500 to 1800 °C. The final material underwent screening and magnetic treatment, yielding MgO with a content of 87–90%. The two-step calcination process involved using natural magnesite through flotation and thermally separating it into the burning furnace. Initially, it was calcined at approximately 1000 °C to produce an L-MgO powder. Subsequently, the powder underwent fine grinding to achieve a specific size and then was then ballasted. The pressed light-burned MgO balls were subjected to vertical shaft kiln calcination at the temperature of 1200–1500 °C. This method can obtain high-quality, medium-grade, and high-purity D-MgO. The research findings indicated that the one-step method required lower thermal energy compared to the latter.^25,26^ Moreover, the purity achieved through the one-step calcination process meets the requirement (e.g., over 85%) for MKPC.^27,28^ Consequently, the one-step calcination process for D-MgO production was studied in this study. Figure 2 illustrates the material consumption, energy and heat consumption, and emissions during the production of D-MgO.^23^ Based on the decomposition of MgCO_3_ and the results of refs (24) and (29), the inventory data for D-MgO were established and are presented in Table S2 in the Supporting Information. Using this data, the process information and corresponding input streams for D-MgO were established in the OpenLCA software from the base streams.

**Figure 3:**
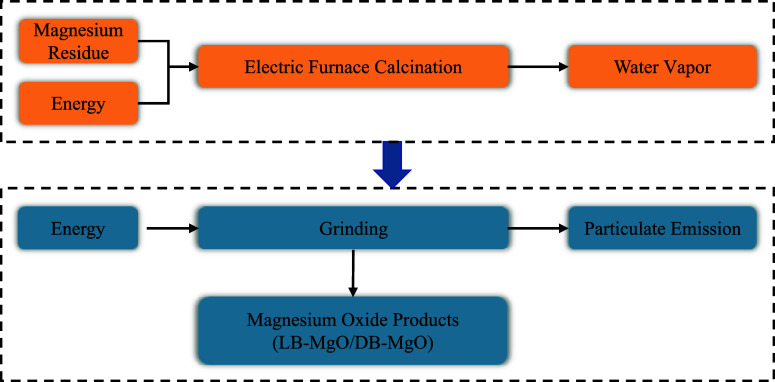
Energy consumption, heat consumption, and emissions during the production of LB-MgO and DB-MgO.

LB-MgO was derived from the Salt Lake magnesium residue, a byproduct of Qarhan Salt Lake brine during the extraction of Li_2_CO_3_, offered by Qinghai CITIC Guoan Co. Ltd. The Salt Lake magnesium residue included 52.3% MgO, 12.69% Mg_3_B_2_O_6_, and 35.05% Mg(OH)_2_ (by mass).^18^ To prepare LB-MgO, the magnesium residue was further calcinated in a high-pressure furnace at a temperature of 600 °C for 2 h.^16^ After calcination, a ball mill was employed to grind the magnesium-rich residue at 45 rad/min for 5 min, achieving a particle size of less than 74 μm. The material, energy, heat consumption, and emissions during the production of LB-MgO are illustrated in Figure 3. Due to the absence of an energy consumption inventory for the calcination of magnesium residue at 600 °C, theoretical calculations for energy consumption during calcination were conducted following the method proposed by Ruan et al.^22^ The heat capacities and enthalpies of individual materials were sourced from the thermochemical tables,^30^ with detailed calculations provided in Table S3 in the Supporting Information. The process data and corresponding input streams were subsequently input into the calculation software based on the inventory data (refer to Table S3 in theSupporting Information).

DB-MgO was derived from the same raw material as LB-MgO. The high Mg(OH)_2_ content in the Salt Lake magnesium residue accelerated the setting speed of MKPC, which showed negative effects on the workability and mechanical properties of MKPC.^18^ To address these challenges, the raw material was further calcinated at 1000 °C for 3 h.^18^ Following calcination, the magnesium-rich residue underwent grinding in a ball mill at 45 rad/min for 5 min, resulting in DB-MgO with an uniform particle size and high purity (approximately 82.63%).^15^ The energy consumption, heat consumption, and emissions during the production of DB-MgO are similar to those of LB-MgO and are depicted in Figure 3. In parallel with LB-MgO, theoretical calculations for the energy consumption during calcination were conducted using the method outlined by Ruan et al.^22^ The detailed calculation process and process inventory for the preparation of DB-MgO are shown in Table S4 in the Supporting Information, from which the process data and corresponding input flow were established.

Four MgO production processes were considered in this study, and Table 1 summarizes the calcination temperatures and energy sources of different kinds of MgO. The primary process employed in clinker production was calcination. In the conventional magnesium industry in China, a vertical shaft kiln was utilized, and coal was the primary fuel. Therefore, the consumption of mineral energy and the environmental impact of coal combustion were considered for L-MgO and D-MgO. As there is a lack of detailed literature on the use of magnesium residue in the byproducts of Salt Lake, the use of high-temperature electric furnaces for calcination was considered, and the energy source was mixed electrical energy.

**Table 1:** Calcination Temperature and Energy Type for Different Types of MgO

type	calcination temperature	type of energy	source	ref.
L-MgO	750–850 °C	coal	magnesite	(31,32)
D-MgO	1500 °C	coal	magnesite	(15)
LB-MgO	600 °C	electricity	The Salt Lake magnesium residue	(18)
DB-MgO	1000 °C	electricity	The Salt Lake magnesium residue	(19)

#### 2.1.2 Other Raw Materials

FA and GGBS were used to replace some of the MgO in the magnesia-based cement. The purity of magnesium sulfate heptahydrate (MgSO_4_·7H_2_O) was 99.0%. Reagent-grade citric acid (CA) as an admixture was used in the BMSC. The raw materials mentioned above were used in the LCA calculations using relevant inventory data from the database.

The neutralization method was used to prepare potassium dihydrogen phosphate (KDP).^33−36^ This method involved using a potassium hydroxide solution neutralized with a thermal-process phosphoric acid, as depicted in
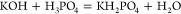
1

The production process included the following procedures. A potassium hydroxide solution (30% KOH) was added to an enamel reactor equipped with stirring and a steam jacket. Subsequently, thermal-processed phosphoric acid (50% H_3_PO_4_ solution) was gradually added under stirring to facilitate the neutralization reaction. Throughout this process, the reaction temperature was maintained between 85 and 100 °C, while the pH value was kept in the range of 4.2–4.6. The reaction end point solution should exhibit a relative density of 1.32–1.33. Following evaporation to achieve a relative density of 1.38–1.42, the solution was directed to the crystallization process. Cooling the solution below 36 °C prompted the precipitation of crystals, which were then subjected to separation, dewatering, washing, and drying stages to produce KDP. This production process, in conjunction with the studies of Lei^37^ and Ping^38^ on KDP production process inventory data, facilitated the establishment of process data and corresponding input streams in the software.

#### 2.1.3 Magnesia-Based Cement

Ten kinds of magnesia-based cement prepared by the above-mentioned four kinds of MgO were studied, and the cement can be divided into three groups, i.e., BMSC, CRMC, and MKPC. The main components of the magnesia-based cement are enumerated in Table 2.

**Table 2:** Definition and Composition of Magnesia-Based Cement^a^

			components of magnesia-based cement										
cement type	definition	raw materials of MgO	D-MgO	DB-MgO	L-MgO	LB-MgO	MgSO_4_·7H_2_O	KDP	FA	GGBS	borax	CA	ref.
BMSC-L	basic magnesium sulfate cement	magnesite			0.682		0.3146					0.0034	(31,32)
BMSC-B	basic magnesium sulfate cement	The Salt Lake magnesium residue and magnesite			0.1263	0.505	0.3655					0.0032	(16)
BMSC-FA	basic magnesium sulfate cement with 17% FA	magnesite			0.5662		0.2611		0.1699			0.0028	(39)
BMSC-S	basic magnesium sulfate cement with 17% GGBS	magnesite			0.5662		0.2611			0.1699		0.0028	(39)
CRMC-L	carbonated reactive magnesia cement	magnesite			1								(40−42)
CRMC-B	carbonated reactive magnesia cement	The Salt Lake magnesium residue				1							
MKPC-D	magnesium potassium phosphate cement	magnesite	0.5935					0.359			0.0475		(15)
MKPC-B	magnesium potassium phosphate cement	The Salt Lake magnesium residue		0.6089				0.3424			0.0487		(15)
MKPC-B-FA	magnesium potassium phosphate cement with 31.7% FA	The Salt Lake magnesium residue		0.4651				0.2179	0.317				(18)
MKPC-B-S	magnesium potassium phosphate cement with 16.7% GGBS	The Salt Lake magnesium residue		0.5674				0.2659		0.1667			(19)

a Note: D-MgO represents dead-burned magnesium oxide produced by magnesite; DB-MgO represents boron-containing dead-burned magnesium oxide produced by the Salt Lake magnesium residue; L-MgO represents light-burned magnesium oxide produced by magnesite; LB-MgO represents boron-containing light-burned magnesium oxide produced by the Salt Lake magnesium residue; MgSO_4_·7H_2_O represents magnesium sulfate heptahydrate; KDP represents potassium dihydrogen phosphate; FA represents fly ash; GGBS represents ground granulated blast-furnace slag; and CA represents analytically pure citric acid.

##### 2.1.3.1 BMSC

The first BMSC in this study was prepared with L-MgO, and the cement was referred to as BMSC-L. The impact of the molar ratio of active magnesia (α-MgO) to MgSO_4_·7H_2_O on the mechanical properties and microstructural development of BMSC-L was examined by Wu^43^ and Tan et al.^44^ The α-MgO content of L-MgO was 60%. The molar ratio of α-MgO/MgSO_4_·7H_2_O was 8:1, and the CA content remained at 0.5% of the mass of L-MgO. This formulation resulted in a production ratio of 1 kg of BMSC-L.

The second BMSC was prepared from LB-MgO and L-MgO, namely, BMSC-B. Wu et al.^16^ explored the feasibility of BMSC utilizing LB-MgO as a clinker, derived from the Salt Lake magnesium residue—a byproduct of the Salt Lake during the Li_2_CO_3_ extraction. Their investigation revealed that substituting 20% of LB-MgO with L-MgO resulted in alkali magnesium sulfate cement with high early (1 day) and long-term (28 days) strength. The LB-MgO possessed an α-MgO content of 65.96%,^16^ maintaining a constant molar ratio of α-MgO/MgSO_4_·7H_2_O at 7:1. A 0.5% CA addition, based on the weight of LB-MgO, was incorporated, and the calculations were performed for 1 kg of BMSC. LB-MgO derived from the calcination of the Salt Lake magnesium residue at 600 °C had no direct carbon emissions (process-based emissions), while the thermal energy required for calcination was lower compared to L-MgO prepared by calcination of magnesite, which had a high potential for energy savings and CO_2_ emission reduction.

The third BMSC was prepared with L-MgO and FA, while the fourth BMSC was prepared with L-MgO and GGBS. These two BMSCs were referred to as BMSC-FA and BMSC-S, respectively. The addition of FA and GGBS cannot only improve the setting time and mechanical properties of BMSC but also achieve economic and environmental benefits.^39^ The clinker used in BMSC-FA and BMSC-S was L-MgO with an α-MgO content of 60%. The molar ratio of α-MgO/MgSO_4_·7H_2_O was 8:1, with CA comprising 0.5% of the mass of L-MgO. Additionally, FA and GGBS were separately added into the mixtures at the ratio of 30% of the L-MgO content by mass, which was 17% of 1 kg cement.

##### 2.1.3.2 CRMC

For CRMC, two magnesium sources were considered: (1) the Salt Lake magnesium residue, which was used to prepare CRMC-B, and (2) magnesite, which was used to prepare CRMC-L. Utilizing the magnesium residue as the raw material for MgO production eliminated direct carbon emissions during the manufacturing process, resulting in a reduction of 1.1 kg of CO_2_ compared to magnesite.^13,14^ The required heat energy was 0.425 kWh (refer to the Supporting Information, Table S3), representing a significant decrease of 63.52% compared to the net reaction enthalpy when using magnesite, which was 1.165 kWh.^13^ These two kinds of MgO can absorb approximately 0.5 kg of CO_2_ per 1 kg of cement through carbonation curing.^10^

##### 2.1.3.3 MKPC

The first and second MKPCs in this study were prepared with D-MgO and DB-MgO, respectively; the MKPCs were referred to as BMSC-D and BMSC-B, respectively. The compositions of MKPC-D and MKPC-B were derived from the research of Tan et al.^15^ According to the study, the molar ratio of D-MgO/KDP or DB-MgO/KDP was maintained at 5:1, with borax constituting 8% of the mass of MgO. Borax served as a commonly employed retarder in MKPC, creating a complex on the surface of MgO that proficiently hindered the hydration of MKPC.

The third MKPC was prepared with DB-MgO and FA, while the fourth BMSC was prepared with DB-MgO and GGBS. These two MKPCs were referred to as MKPC-B-FA and MKPC-B-S, respectively. Adding FA, a byproduct of coal combustion, and/or GGBS, generated during ironmaking in blast furnaces, can reduce the consumption of cement clinker, thereby lowering energy consumption and carbon emissions.^45−50^ To reduce the environmental effects of MKPC and improve the properties of MKPC, Tan et al.^18,19^ replaced a part of the DB-MgO in MKPC with FA and GGBS, respectively. The molar ratios of DB-MgO/KDP and the water-cement ratios of MKPC-B-FA were 6 and 0.16, respectively. The dosage of FA was 40% of the DB-MgO and KDP by mass, which was 31.7% of 1 kg of cement. MKPC-B-S had a similar molar ratio of DB-MgO to KDP at 6. The GGBS was added to the cement at the ratio of 20% of B-MgO and KDP by weight, which was 16.7% of 1 kg of cement.

### 2.2 LCA Methodology

#### 2.2.1 Functional Unit and System Boundary

**Figure 4:**
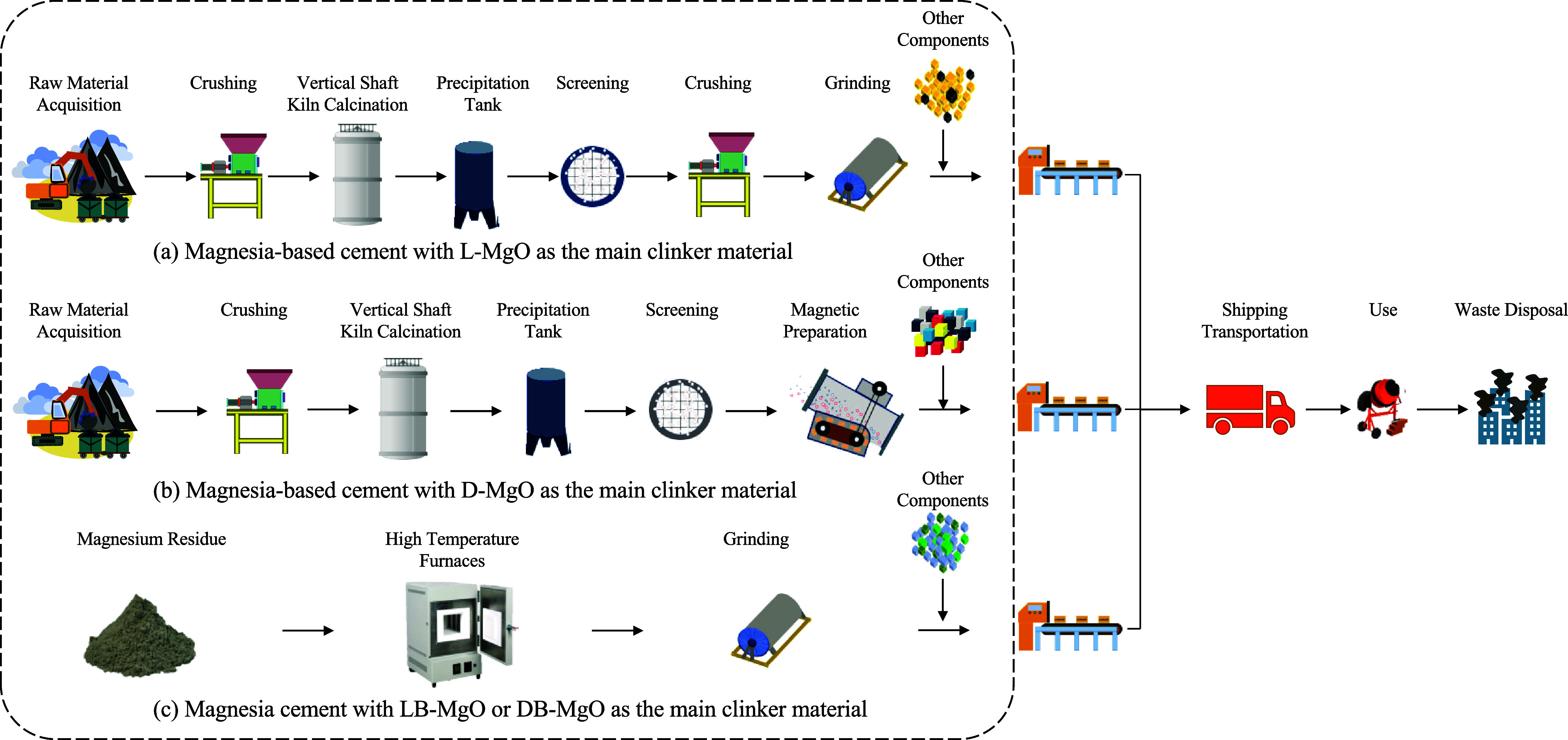
Assessment scope of different kinds of magnesia-based cement, as adapted from ref (23).

Due to the absence of technical standards for various magnesia-based cement products (e.g., mortar or concrete), this study adopted a functional unit of 1 kg for each cement. The environmental effects of the procedure from the raw material acquisition (cradle) to the final product (gate) were analyzed in this study, as illustrated in Figure 4.^23^ This study employed the LCA methodology to scrutinize the diverse inputs and outputs associated with different types of cement. The inputs included energy sources (e.g., electricity, coal, and diesel fuel) and raw materials (e.g., magnesite, magnesium residue, and water), while the outputs involved pollutants and emissions, such as CO_2_, CO, SO_2_, NO_*x*_, and particulate matter. As magnesia-based cement included many components (e.g., MgO, magnesium sulfate heptahydrate, admixtures, etc.), the performance of the final product would be adversely affected by the storage time if all of the components were mixed and then packaged,^43^ while there were too many uncertainties if the components were packed separately. Therefore, the environmental impact of the packaging of the cement products was not evaluated in this study.

#### 2.2.2 Inventory Analysis

In this study, an open-access software, OpenLCA (version 2.0),^51^ was used for modeling and calculating the production of each type of magnesia-based cement based on the composition outlined in Table 2. In addition, generalized process data were sourced from the Swiss Ecoinvent database.^52^ Given that the data for MgO in this study were obtained from some industries in China (Section 2.1.1), this study mainly considered the environmental effects of magnesia-based cement in China. Therefore, the following modeling process also used energy data from China. If Chinese data were unavailable, global or European data were employed as substitutes. Chinese electricity generation primarily relies on thermal power (58.4%), hydropower (15.5%), wind power (8.8%), nuclear power (4.8%), and solar power (4.9%),^53^ while some European countries such as the United Kingdom use a lot of wind power.^54−57^ To consider the diversity of regions and energy sources, a blend of electricity from an electricity supply company was deliberately chosen as the electricity input in this study. The inputs and outputs of all relevant analyses in the Life Cycle Inventory (LCI) and Impact Assessment (IA) are linked to the defined functional units described above.

#### 2.2.3 Impact Assessment

**Figure 5:**
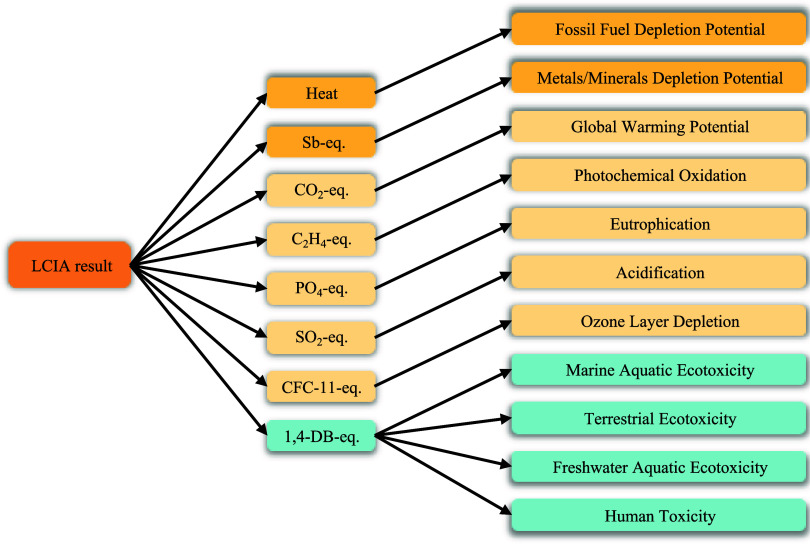
Evaluation process of the CML-IA baseline evaluation methodology. Note: Sb-eq represents antimony equivalent, C_2_H_4_-eq represents ethylene equivalent, PO_4_-eq represents phosphate equivalent, SO_2_-eq represents sulfur dioxide equivalent, CFC-11-eq represents freon-11 equiv, and 1,4-DB-eq represents 1,4-dibutoxybenzene equivalent.

During the analysis of the environmental effects of cement production, utilizing midpoint impacts is better to conduct a comprehensive evaluation of the diverse environmental effects of raw materials and energy during the production process.^58^ In this study, the CML-IA baseline (Institute of Environmental Sciences, Leiden University)^59−62^ midpoint assessment method was employed for the calculations, and Figure 5 delineates the assessment process. The environmental impact categories included global warming potential (GWP100a, measured in CO_2_-eq), fossil fuel depletion potential (FDP, measured in MJ), metal/mineral resource depletion potential (MDP, measured in Sb-eq), acidification potential (AP, measured in SO_2_-eq), eutrophication potential (EP, measured in PO_4_-eq), marine ecotoxicity potential (METP, measured in 1,4-DB-eq), terrestrial ecotoxicity potential (TETP, measured in 1,4-DB-eq), freshwater ecotoxicity (FETP, measured in 1,4-DB-eq), human toxicity potential (HTP, measured in 1,4-DB-eq), ozone depletion potential (ODP, measured in CFC-11-eq), and photochemical ozone creation potential (POCP, measured in C_2_H_4_-eq).

The magnesia-based cement in this study was standardized and weighted using the EDIP2003 method^63,64^ to derive the Overall Environmental Impact Indicator (OEI). When magnesia-based cement products were evaluated, their contribution to the environmental impacts was discussed from the following two perspectives.

(a) The direct contribution was the process-based emissions referred to emissions from chemical or production processes, including consumption and emissions in the production process of cement clinker and other components (such as CA, KDP, magnesium sulfate heptahydrate, etc.) and emissions from energy utilization.
(b) Indirect contribution referred to energy production (such as coal, diesel, electricity, etc.) and the acquisition of raw materials, consumption, and emissions during transportation.

#### 2.2.4 Sensitivity Analysis

Determining the level of uncertainty is important in the LCA analysis. Typically, the uncertainty of LCIA results is usually caused by the limitations and inaccuracies of the study checklist parameters and calculation models,^65,66^ and these levels of uncertainty can be determined by sensitivity analysis.^67^ In this study, the impact of different parameters on LCIA results was examined by analyzing model input parameter variations within a range of ±15%, as suggested by refs (10) and (68). The Monte Carlo method was employed for 1000 simulations, utilizing a uniform distribution as the distribution function for the input parameters. The study focused on the variation in raw materials and mineral energy consumption during L-MgO and D-MgO production, considering the diversity of raw materials, energy, and equipment. Additionally, there were also differences in the percentage of MgO and Mg(OH)_2_ content in different batches of the Salt Lake magnesium residue, which resulted in inconsistency in the heat required at the calcination stage. This, in turn, led to different power consumptions in the high-temperature electric furnaces, so fluctuations in the electricity consumption required for the production of LB-MgO and DB-MgO were considered. Furthermore, the inventory data of KDP came from the retrieval and theoretical calculations of the existing literature, which also caused uncertainty; therefore, the fluctuation of the inventory parameters of KDP was mainly examined.

## 3 Results and Discussion

### 3.1 CO_2_ Emissions of MgO

**Figure 6:**
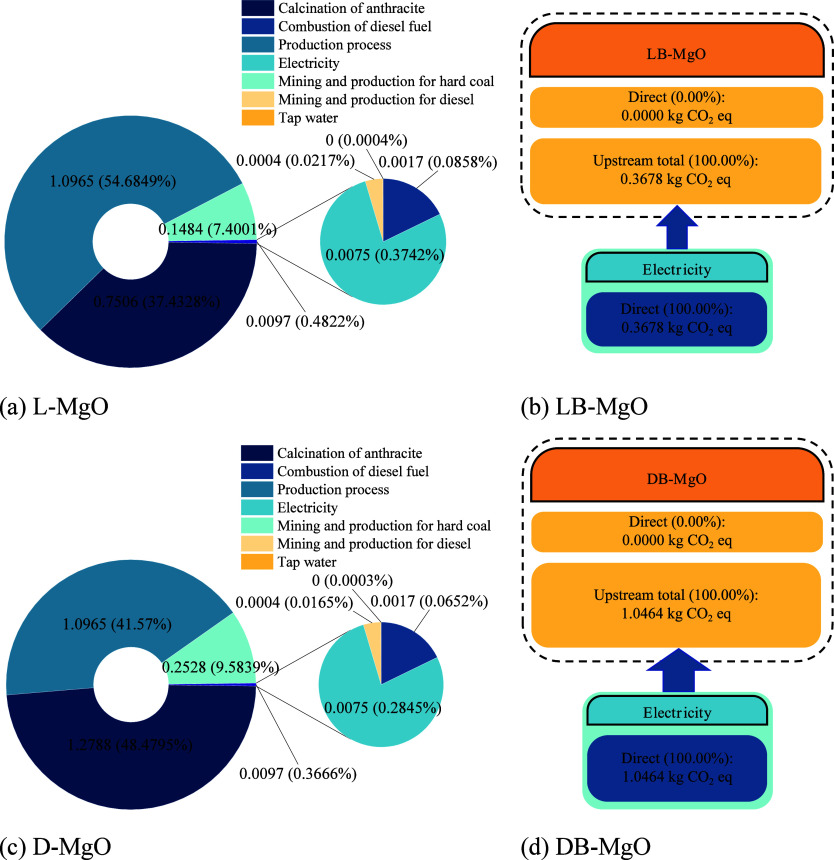
CO_2_ emissions of different types of MgO.

The CO_2_ emissions of four MgO products (i.e., L-MgO, LB-MgO, D-MgO, and DB-MgO) were evaluated, and the results are presented in Figure 6. The carbon footprint of L-MgO was 2.01 kg of CO_2_-eq (Figure 6a). Specifically, the production process accounted for 54.7% of the total carbon emissions, while the figure for heat demand (combustion of anthracite) was 37.4%. The proportion of the carbon emissions due to the mining and production of hard coal was 7.4%, while only 0.5% of the total CO_2_ emissions came from electricity production, diesel extraction and production, diesel combustion, and tap water production. The carbon footprint of D-MgO was 2.64 kg of CO_2_-eq/kg (Figure 6c), which was higher than that of L-MgO (Figure 6c). Due to the high-temperature demand (1500 °C) during the calcination, up to 48.5% of total CO_2_ emissions of D-MgO came from coal combustion. The CO_2_ emissions during the production processes were the second highest, at 41.6%, and the hard coal mining and production accounted for 9.6%. These results are in good agreement with the results of An and Xue,^25^ which showed that the carbon footprint of L-MgO was 1.6–1.8 kg CO_2_-eq/kg, and the figure for sintered magnesium oxide was 2.0–2.7 kg CO_2_-eq/kg. However, the carbon footprint assessment in this study was more comprehensive than ref (26) because it did not consider the carbon emissions from magnesite mining. In addition, the amounts of indirect CO_2_ emissions (i.e., due to power production) of LB-MgO and DB-MgO were 0.37 and 1.05 kg CO_2_-eq/kg, respectively (Figure 6b,d), which were 81.7 and 60.3% lower than those of L-MgO and D-MgO, respectively. This means that using the MgO derived from the Salt Lake residues is more environmentally friendly compared with traditional MgO.

### 3.2 CO_2_ Emissions of Magnesia-Based Cement

**Figure 7:**
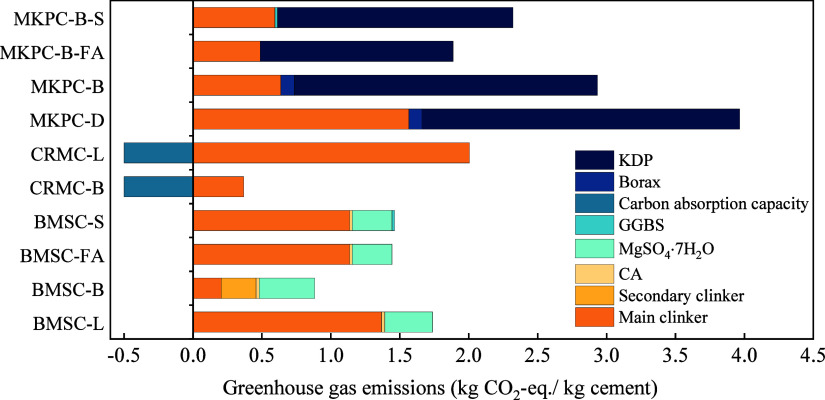
CO_2_ emissions of different types of magnesia-based cement Notes. The main and secondary clinkers in BMSC-B are LB-MgO and L-MgO, respectively.

Figure 7 further illustrates the carbon footprints of various magnesia-based cement. The total carbon emissions of CRMC-L and CRMC-B were 2.01 and 0.37 kg of CO_2_-eq/kg, respectively, and CRMC-B contributed the lowest carbon emissions among all the magnesia-based cement. In this study, it was assumed that 1 kg of CRMC can absorb 0.5 kg of CO_2_ by carbonation because the reactive MgO in CRMC can absorb large amounts of carbon dioxide during the carbonation stage.^21,31,32,69,70^ Consequently, the net CO_2_-eq values for CRMC-L and CRMC-B were 1.51 and −0.13 kg, respectively. This means that over 100% CO_2_ emissions reductions can be achieved if LB-MgO was used to produce CRMC. Ruan et al.^22^ investigated the feasibility of using waste brine from desalination plants to produce CRMC and found that when nuclear energy, natural gas, and coal were used to produce electricity, the carbon footprints of CRMC were 1.76, 2.32, and 4.56 kg CO_2_-eq/kg, respectively. Ruan et al.’s results were much higher than this study, which means that using the Salt Lake magnesium residue to prepare MgO for CRMC is an environmentally friendly approach.

In the group of MKPCs, MKPC-D utilizing D-MgO as clinker was the highest emitter with 3.97 kg of CO_2_-eq. The primary contributors to this carbon footprint were the manufacturing of KDP and D-MgO. The substitution of D-MgO with alternative materials such as DB-MgO, FA, and GGBS demonstrated a commendable ability to reduce carbon emissions. Specifically, MKPC-B, MKPC-B-S, and MKPC-B-FA exhibited significant reductions in CO_2_ equivalent by 26.04, 41.47, and 52.44%, respectively, compared to MKPC-D. The carbon emissions of MKPCs were mainly caused by the production of KDP through the neutralization method,^33−36,71−73^ which was an energy-intensive technology. Excluding the energy consumption from thermal-process phosphoric acid and potassium hydroxide, it was estimated that approximately 1.2 tons of coal and 200 kWh of electricity were required to produce 1 ton of KDP.^37,38^ Therefore, exploring alternative materials and optimizing energy-intensive processes, particularly in the production of key components such as KDP, are crucial to achieving the net zero target in MKPC-D.

In comparison to MKPCs, BMSCs exhibited lower-carbon emissions. BMSC-B had the lowest carbon emissions of the four BMSCs, at 0.88 kg of CO_2_-eq/kg. The substitution of LB-MgO for a portion of L-MgO demonstrated remarkable energy savings and the reduction capability of carbon emissions, resulting in a 49.22% decrease compared to BMSC-L (0.88 kg of CO_2_-eq vs 1.60 kg of CO_2_-eq). Similarly, using FA and GGBS to replace a portion of the clinker contributed to a reduction in carbon emissions by 16.99% (1.46 kg CO_2_-eq vs 1.60 kg CO_2_-eq) and 15.85% (1.44 kg CO_2_-eq vs 1.60 kg CO_2_-eq), respectively.

### 3.3 Fossil Fuel Depletion Potential

Given the variation in fossil fuel and raw material requirements across different types of magnesia-based cement, cumulative energy demand was utilized to quantify the fossil energy needs per unit mass (MJ/kg) of the cement.

**Figure 8:**
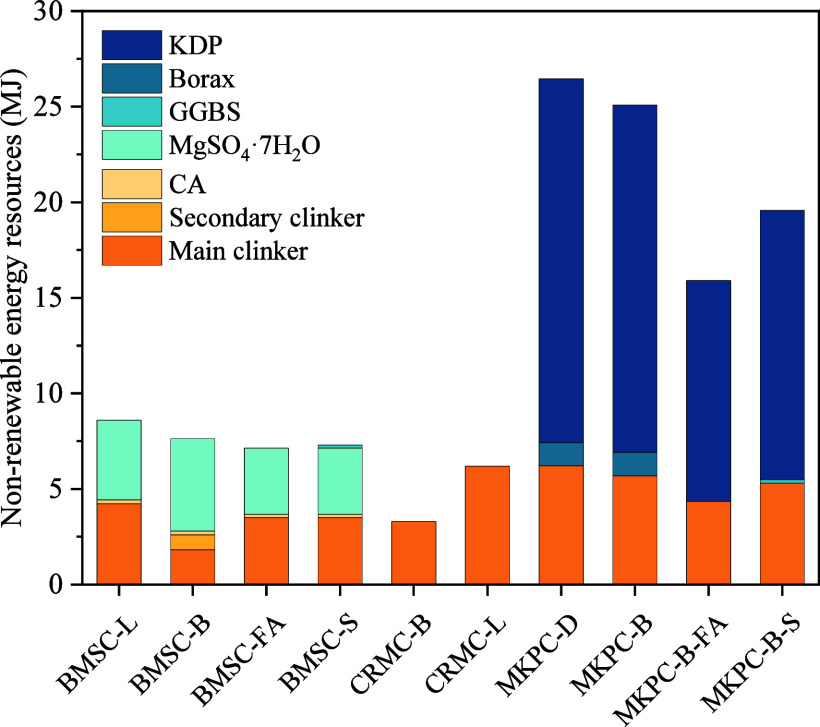
Fossil fuel depletion potential of various magnesia-based cement.

Figure 8 presents the fossil energy depletion potential of magnesia-based cement. CRMC-B exhibited the lowest cumulative energy demand at 3.29 MJ/kg, which can be attributed to its reduced power consumption during the calcination process. On the contrary, the cumulative energy demands of MKPC-D and MKPC-B ranked the first and second highest, at 26.45 and 25.08 MJ/kg, respectively. The distributions of cumulative energy demand between these two kinds of cement were notably similar. KDP accounted for 71.95 and 72.39% of the total fossil energy depletion potentials of MKPC-D and MKPC-B, respectively, while the figures for D-MgO were 23.52 and 22.71%, respectively. On the contrary, the contribution of CA in MKPC-D and MKPC-B was smaller at 0.05%.

The cumulative energy demand for BMSC-L, BMSC-B, BMSC-FA, and BMSC-S was 8.59, 7.64, 7.13, and 7.23 MJ/kg, respectively, which can mainly be ascribed to the utilization of magnesium sulfate heptahydrate and MgO. In addition, substituting L-MgO with LB-MgO, FA, and GGBS reduced the cumulative energy demand by 11.12, 17.01, and 15.07%, respectively.

Table 3 lists the fossil fuel depletion potentials of L-MgO and D-MgO. For CRMC-L prepared with L-MgO, the cumulative energy demand was 6.19 MJ/kg, primarily stemming from its calcination process. This aligned closely with the findings of ref (74): the theoretical energy demand for L-MgO production via magnesite calcination was 5.9 MJ/kg. Additionally, the actual heat demand for D-MgO during calcination was 10.39 MJ/kg, while the theoretical heat requirement for D-MgO calcined at a high temperature of 1500 °C was 6.68 MJ/kg (refer to Table S2 in the Supporting Information). Industrial production of D-MgO using a rotary kiln exhibited a significant heat loss of approximately 37%.^75,76^ Consequently, the theoretical energy demand, including heat loss, was estimated at 10.6 MJ/kg, demonstrating the results in this study. Therefore, the magnesium industry should pay closer attention to enhancing the heat utilization efficiency during production.

**Table 3:** Fossil Fuel Depletion Potential of L-MgO and D-MgO (KJ)

source	L-MgO	D-MgO
electricity	67.07	67.07
coal	6098.59	10,390.19
diesel	27.99	27.99
tap water	0.09	0.09
total	6193.73	10,485.33

### 3.4 Other Environmental Impacts

**Figure 9:**
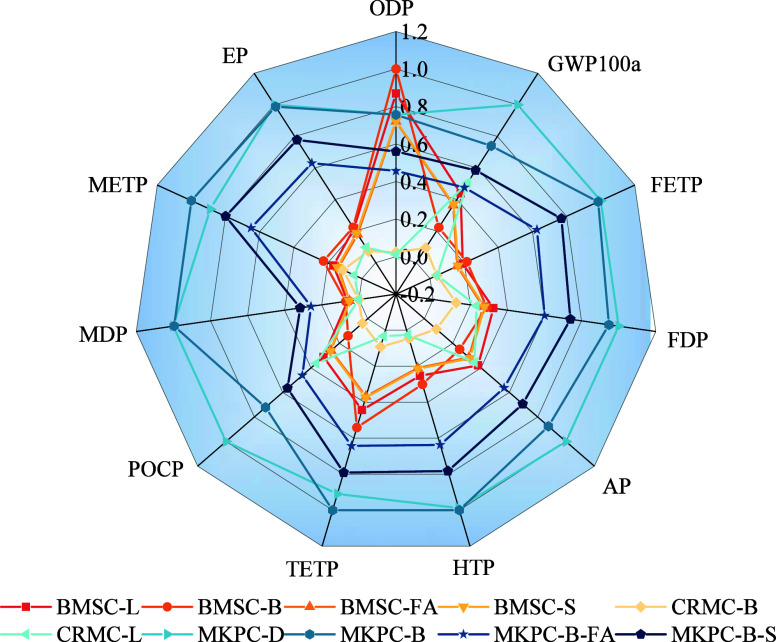
Normalized impact categories.

**Figure 10:**
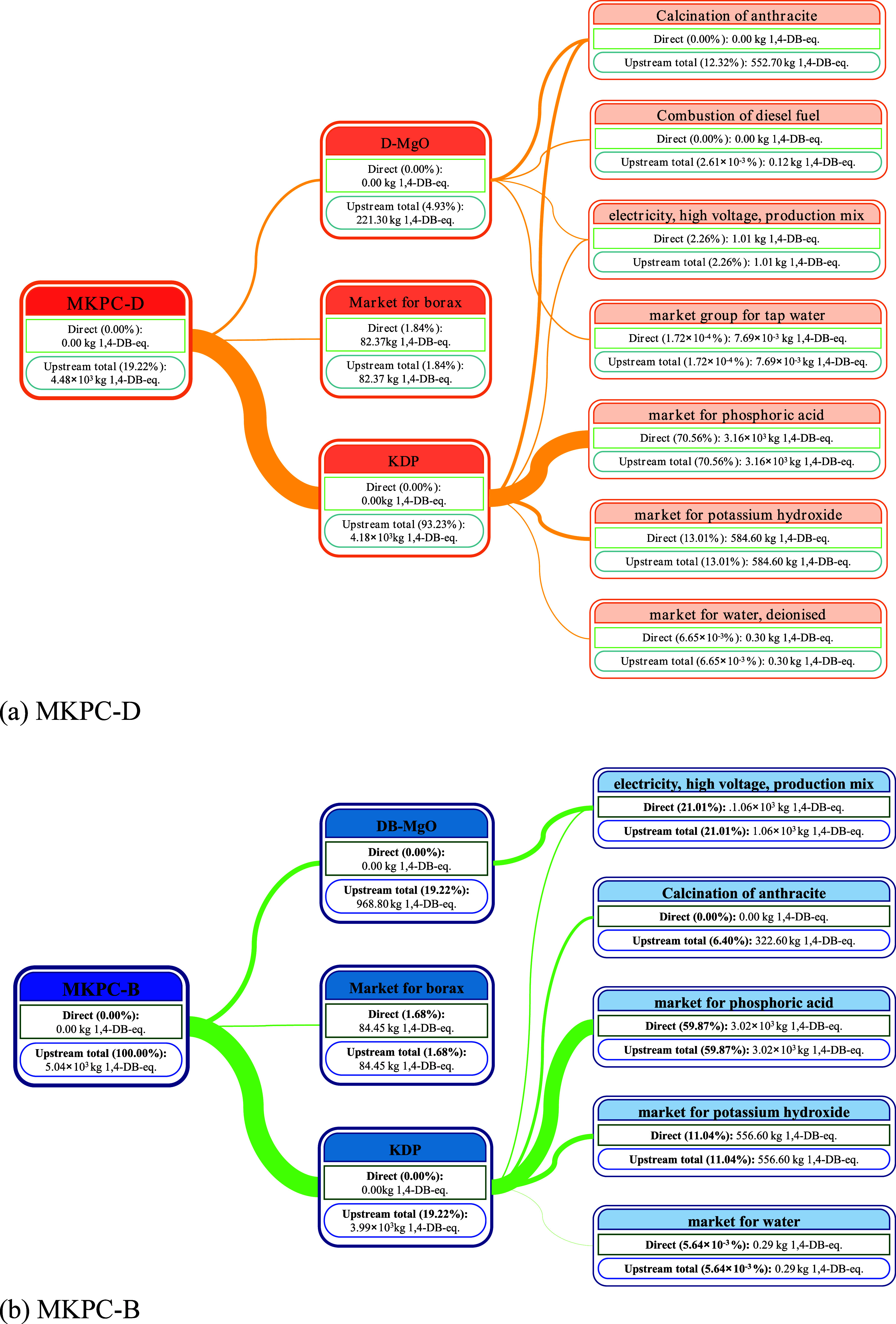
Sankey diagram of marine ecotoxicity potential.

Figure 9 illustrates the normalized LCA results. The maximum value of the 11 environmental impact indicator values was set to 100%, and the remaining values were varied accordingly. Compared with BMSC-L, CRMC-L, and MKPC-D, their alternatives (i.e., BMSC-B, BMSC-FA, BMSC-S, CRMC-B, MKPC-B, MKPC-FA, and MKPC-S) consistently exhibited lower impacts in terms of certain environmental impacts. Notably, CRMC-B was the top performer across all categories, attributed to its low-carbon production process, which significantly minimized its contribution to all environmental indicator values. Moreover, MKPC-D exhibited higher indicator values across all 10 environmental impacts, except for ozone layer depletion. Notably, six indicators (GWP100a, FETP, ADP, AP, POCP, and MDP) demonstrated the highest value. Conversely, MKPC-B surpassed MKPC-D and attained the highest values in HTP, TETP, and METP. MKPC-B was particularly significant in METP. Figure 10 shows a Sankey diagram of the METP of MKPC-D and MKPC-B, showing the 1,4-DB-eq produced at various stages of their production. The METP indicator for MKPC-D and MKPC-B was 4484.52 kg 1,4-DB equiv and 5040.83 kg 1,4-DB eq, respectively. For MKPC-D, thermal-process phosphoric acid, potassium hydroxide, and coal mining and production contributed 70.56, 13.01, and 12.32%, respectively. In contrast, for MKPC-B, the acquisition and production of raw materials of thermal-process phosphoric acid contributed 59.87%; the production of electricity, another major contributor to high METP due to excessive electricity consumption for high-temperature calcination (1000 °C) of DB-MgO, contributed 21.01%, and the acquisition and production of raw materials of potassium hydroxide contributed 11.04%. The METP of MKPC-B-FA and MKPC-B-S was lower than that of MKPC-D by 26.91 and 20.08%, respectively, but it was higher than those of BMSCs and CRMCs. In conclusion, it can be found that the clinker and minor component KDP in MKPC have a significant adverse effect on the flora, fauna, and human health in the natural environment.

All of the BMSCs exhibited elevated ODP indicators, and BMSC-B showed the highest values. This is primarily ascribed to the production of magnesium sulfate heptahydrate, a process associated with the substantial generation of equivalent CFC-11 emissions, contributing significantly to the severe depletion of the ozone layer.

### 3.5 Overall Environmental Impact Indicators

Weighting is an optional step in the ISO 14044 requirements^77^; it involves using numerical factors derived from value selection to facilitate comparisons between impact category indicators (or standardized results).^78^ This entails standardizing the results of characterizing all categories, multiplying them by a weighting factor, and subsequently summing them to derive an Overall Environmental Impact Indicator or Single Score. In this study, a lower single score signified a reduced environmental burden, indicating a more positive overall environmental impact and greater favorability toward the environment.

**Figure 11:**
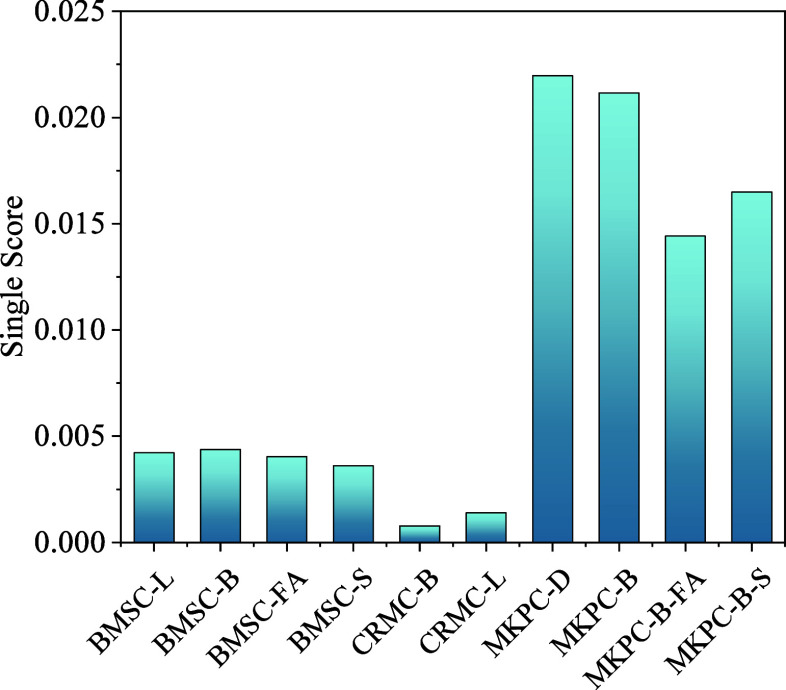
Single score for various magnesia-based cement.

In this study, 10 types of magnesia-based cements were studied using the EDIP2003 method, and the individual scores are presented in Figure 11. Notably, CRMC-B achieved a score of 0.00078, which means that CRMC-B showed the lowest environmental burden. In addition, among the four BMSCs, BMSC-B exhibited a slightly higher score than BMSC-L. This implies that the substitution of LB-MgO for the majority of L-MgO did not show a distinct advantage in terms of the overall environmental impact. However, the incorporation of GGBS or FA into BMSC was found to yield favorable outcomes in terms of overall environmental benefits. Moreover, MKPC-D showed the highest overall environmental impact index with a score of 0.02197. MKPC-B, MKPC-B-S, and MKPC-B-FA exhibited varying degrees of reduction compared to MKPC-D. This means that incorporating DB-MgO in MKPC was environmentally friendly. Replacing D-MgO while doping GGBS or FA can greatly reduce the overall environmental impact of MKPC.

### 3.6 Sensitivity Analysis

Monte Carlo simulation calculations were utilized to conduct a sensitivity analysis on magnesia-based cement, and the detailed results are provided in Tables S6–S15 in the Supporting Information. Table 4 lists the maximum and minimum coefficients of variation of magnesia-based cement. The results reveal that when the input parameters with inherent uncertainties fluctuated within the range of ±15%, the coefficient of variation of the environmental impact indicators of 10 kinds of magnesium-based cement varied from 0.06 to 8.76%. The smaller the coefficient of variation, the smaller the difference between the data points obtained by Monte Carlo simulation, and the higher the reliability of the data set. Notably, the smallest impact range, from 0.06 to 2.60%, was observed for BMSC-B. This can be attributed to the fewer parameters considered for uncertainties in this cement (only the uncertainty of B-MgO was considered), resulting in lower sensitivity to various environmental impacts. Conversely, CRMC-B exhibited the widest impact variation range, reaching 8.76% for all environmental impact categories. This significant variation was primarily attributed to the influence of fluctuations in input electrical energy, leading to a broader spectrum of changes in overall environmental impacts. The coefficient of variation of MKPC-B-FA and MKPC-B-S was also large, primarily attributed to the consideration of multiple uncertainties (LB-MgO and KDP). In addition, the coefficients of variation for the environmental impact indicators of the 10 kinds of magnesia-based cement in this study ranged from 0.06 to 8.76% (Table 4), which was smaller than 10% as suggested in refs (79) and (80). Therefore, the LCIA results obtained under the assumptions and uncertainties of this study possessed a reasonable level of credibility.

**Table 4:** Maximum and Minimum Coefficient of Variation of Magnesia-Based Cement

cement	min	max	cement	min	max
BMSC-L	0.07%	4.96%	BMSC-B	0.06%	2.60%
BMSC-FA	0.07%	5.15%	BMSC-S	0.07%	4.86%
CRMC-B	8.76%	8.76%	CRMC-L	6.12%	8.56%
MKPC-D	3.21%	6.64%	MKPC-B	3.03%	6.33%
MKPC-B-FA	4.52%	7.75%	MKPC-B-S	4.50%	7.68%

**Figure 12:**
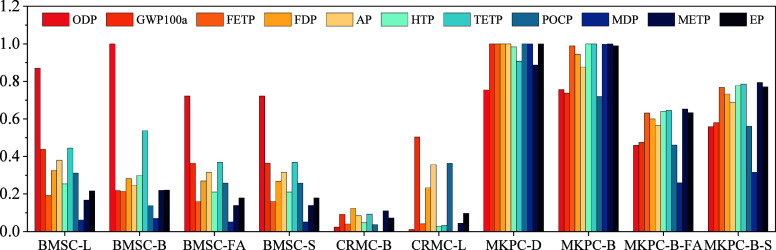
Single score for magnesia-based cement.

Figure 12 shows the results of normalization of the average values of 11 environmental impact indicators for 10 types of magnesia-based cement under Monte Carlo simulation. MKPC-D and MKPC-B presented the largest environmental burden. MKPC-D had seven normalized results of 1, which showed that its negative impact on the environment was the biggest; MKPC-B had three normalized results of 1, and its negative impact on the environment was in second place. The impact of CRMC-B on the 11 environmental categories was the smallest, in which the largest normalized result was FDP with a value of 0.12, which was 12.35% of that of MKPC-D. This means that the preparation of CRMC from the Salt Lake magnesium residue was a green and feasible solution. In addition, manufacturing all BMSCs significantly consumed the ozone layer compared with other magnesium-based cement. The ODP normalized value was 1 for BMSC-B and 87.07, 72.22, and 72.21% for BMSC-L, BMSC-FA, and BMSC-S, respectively.

## 4 Conclusions

This paper conducted a life cycle assessment to compare the environmental impacts of three major groups of magnesia-based cement (i.e., BMSC, MKPC and CRMC). The impacts included the global warming potential, fossil fuel depletion potential, and overall environmental impact indicators. To gauge the level of uncertainty in the life cycle assessment results, sensitivity analyses were conducted on model input parameters with substantial uncertainties. The following key conclusions can be drawn:

(1) Utilizing the Salt Lak magnesium residue to produce light-burned MgO and dead-burned MgO presented significant environmental advantages compared to traditional magnesite mining methods. This approach not only substantially reduced the carbon emissions of MgO (81.66% and 60.33% CO_2_-eq/kg compared to L-MgO and D-MgO, respectively) but also addressed the challenge of managing magnesium residue generated in the Li_2_CO_3_ industry. Developing cleaner energy sources in MgO production was anticipated to further decrease the environmental impact of MgO products.
(2) The incorporation of FA and GGBS into BMSC and MKPC resulted in varying reduction degrees of carbon emissions (16.99 and 15.85% for BMSC, respectively; 41.47 and 52.44% for MKPC, respectively). The CRMC prepared with LB-MgO was a promising option to achieve the net zero target of magnesia-based cement.
(3) For MKPC, the substantial heat demand primarily came from the calcination process of dead-burned MgO and the production of KDP. This resulted in considerable consumption of fossil and electrical energy, raising significant environmental concerns.
(4) From an analysis of the overall environmental impact indicators, the ranking of the 10 magnesia-based cement was as follows: MKPC-D > MKPC-B > MKPC-B-S > MKPC-B-FA > BMSC-B > BMSC-L > BMSC-FA > BMSC-S > CRMC-L > CRMC-B. MKPC-D was the least environmentally sustainable material among the above magnesium cementitious options, whereas CRMC-B was an environmentally friendly cementitious material.
(5) The sensitivity analysis conducted shed light on the uncertainties associated with input parameters in the model. The results demonstrated that even with fluctuations of up to ±15% in the model’s input parameters, the impacts on the LCA results remained within acceptable limits based on the assumptions and uncertainties addressed in this study. This signified that the model outputs maintain a relatively stable profile, even considering parameter uncertainties.

The adverse environmental impacts of magnesia-based cement mainly originate from the production process of MgO. Future studies should study MgO prepared with lower-carbon magnesium sources and improve the energy utilization efficiency of the calcination process.

## Supporting Information Available

The detailed inventory of the four kinds of MgO and potassium dihydrogen phosphate, the theoretical thermal energy calculation results, the sensitivity analysis results, and relevant references (PDF)
