# Supplementary material for: Is It Possible to Make Magnesia-Based Cement Environmentally Friendly?
Source: ACS Sustain Chem Eng. 2024 Nov 6;12(46):16869–83. doi: 10.1021/acssuschemeng.4c05985 (PMC11577429; doi:10.1021/acssuschemeng.4c05985)
Supplement: Supplementary file 1 — sc4c05985_si_001.pdf [file sc4c05985_si_001.pdf]

Is it possible to make magnesia-based cement environmentally friendly?

Yongshan Tan [a, *], Shichang Liu [a], Mithila Achintha [b], Renjie Mi [c, *]

[a] College of Civil Science and Engineering, Yangzhou University, Yangzhou, 225127, China
[b] School of Engineering, The University of Manchester, Oxford Road, Manchester, M13 9PL, United Kingdom
[c] Department of Engineering, University of Cambridge, Cambridge, CB3 0FA, United Kingdom

* Corresponding author. Email: ystan@nuaa.edu.cn

* Corresponding author. Emails: rm2175@cam.ac.uk, renjiemi3-c@my.cityu.edu.hk

Number of pages: 11
Number of tables: 15

This supplementary data contains the detailed inventory of the four kinds of MgO and potassium dihydrogen phosphate, the theoretical thermal energy calculation results, and the sensitivity analysis results.

S1. Inventory for the production of L-MgO from magnesite

The inventory for producing 1 ton of L-MgO by magnesite was summarized by the Ref. [1] and is listed in Table S1.

Table S1 Inventory for the production of 1 ton of L-MgO [1]

Input/emissions		Amount
Raw materials	Magnesite (ton)	2.17
	Water (ton)	0.01
Energy	Hard coal (ton)	0.27
	Electricity (kWh)	8.67
	Diesel (kg)	0.54
Emissions	CO_2 (kg)	1096.54
	SO_2 (kg)	4.21
	CO (kg)	0.02
	NO_x (kg)	0.01
	Water vapor (ton)	0.01
	Magnesite dust (kg)	0.99

S2. Theoretical thermal energy consumption and inventory for the production of D-MgO from magnesite

The theoretical thermal energy for D-MgO can be calculated as follows.

The heat absorption of magnesite decomposition:

$$Q_1 = \Delta H = \Delta_{298}^{\Theta} = (-393.52) + (-601.24) - (-1111.69) = \text{ 116.93 kJ/kg}$$

The amount of heat required to heat CO_2:

$$Q_2 = \int_{298}^{1773} C_P \mathrm{d}T = \int_{298}^{1773} (44.14 + 9.04\times10^{-3}T - 8.54\times10^{5}/T^{2})\mathrm{d}T = \text{ 76529.75 kg/k mol} = \text{76.53 kJ/mol}$$

The amount of heat required to heat MgO:

$$Q_3 = \int_{298}^{1773} C_P \mathrm{d}T = \int_{298}^{1773} (48.99 + 3.14\times10^{-3}T - 11.72\times10^{5}/T^{2})\mathrm{d}T = \text{ 73784.32 kJ/kg} = \text{73.78mol kJ/mol}$$

The theoretical power consumption:

$Q_{total} = Q_1 + Q_2 + Q_3 =$ 116.93 kJ/mol + 76.53 kJ/mol + 73.78 kJ/mol = 267.24 kJ/mol = 6681 kJ/kg =

1855.83 kWh/t

The inventory for producing 1 ton of D-MgO by magnesite was summarized by the Refs. [1, 2] and is listed in Table S2.

Table S2 Inventory of the production of 1 ton of D-MgO [1, 2]

Inputs/Emissions		Amount
Raw materials	Magnesite (ton)	2.17
	Water (ton)	0.01
Energy	Hard coal (ton)	0.49
	Electricity (kWh)	8.67
	Diesel (kg)	0.54
Emissions	CO_2 (kg)	1096.54
	SO_2 (kg)	4.21
	CO (kg)	0.02
	NO_x (kg)	0.01
	Water vapor (ton)	0.01
	Magnesite dust (kg)	0.99

S3. Inventory of the production of LB-MgO from magnesium residue

The theoretical calorific value of magnesium residue, subjected to calcination and decomposition at 600 ℃, comprised three primary components. Q_1 was the heat absorption during the decomposition of 1.121 kg of magnesium residue, where $Mg(OH)_2$ transformed into 1 kg of magnesium oxide and 0.121 kg of water at 25 ℃ (298 K), as expressed in

$$Mg(OH)_2 = MgO + H_2O \quad (1)$$

Q_2 was the amount of heat needed to elevate the temperature of the MgO from 25 °C (298 K) to 600 °C (873 K). Q_3 was the heat required to raise the temperature of H_2O from 25 °C to 600 °C.

The total calorific value (Q_{total}) was the sum of Q_1, Q_2, and Q_3. Q_{total} represented the overall heat absorption during the calcination process of 1.121 kg of magnesium residue, resulting in the production of 1 kg of LB-MgO.

The heat absorption of $Mg(OH)_2$ to form 1 mol of MgO at 25 °C (298 K):

$$\Delta H = \Delta_{298}^{\Theta} = (-285.83) + (-601.24) + (-924.66) = 37.593 \text{ kJ/mol} = 939.825 \text{ kJ/kg}$$

$$Q_1 = 939.825 \text{ kJ/kg} \times 1.121 \text{ kg} \times 35.05\% = 369.27 \text{ kJ}$$

The heat required to heat MgO:

$$\int_{298}^{873} (48.99 + 3.14 \times 10^{-3} \cdot T - 11.72 \times 10^{5} / T^{2}) \mathrm{d}T = 26.60 \text{ kJ/mol} = 665 \text{ kJ/kg}$$

$$Q_2 = 665 \text{ kJ/kg} \times (1.121 \times 35.05\% \times 40/58 + 1.121 \times 52.25\%) = 569.70 \text{ kJ}$$

The heat required to heat H_2O:

$$\int_{298}^{873}(-203.60+1523.29\cdot T-3193.41\cdot T^2+2474.45\cdot T^3+3.86/T^2)\mathrm{d}T = \text{ 78.70 kJ/mol} = \text{23900 kJ/kg}$$

Q_3 = 4370 kJ/kg × 1.121 kg × 35.05% × 10/58 = 532.87 kJ

The theoretical power consumption:

$$Q_{total} = Q_1 + Q_2 + Q_3 = \text{ 369.27 kJ + 569.70 kJ + 532.87 kJ = 1471.84 kJ = 0.409kWh}$$

The study [3] suggested that the energy consumption associated with ball milling ranged from 10-16 kWh per ton clinker. In this study, the maximum value in this range was selected to make a conservative estimation, which means the energy consumption for ball milling 1 kg of clinker was 0.016 kWh. Consequently, the comprehensive energy consumption for producing 1 kg of LB-MgO from magnesium residue was established at 0.425 kWh, as shown in Table S3.

Table S3 Inventory of production for 1 kg LB-MgO

Inputs/Emissions		Amount
Raw materials	Magnesium residue (kg)	1.121
Energy	Electricity (kWh)	0.425
Emissions	Water vapor (kg)	0.121

S4. Inventory of the production of DB-MgO from magnesium residue

The theoretical heat absorption calculation of DB-MgO was similar with that for LB-MgO, but the calcination and decomposition temperature was 1000 ℃.

The heat absorption of $Mg(OH)_2$ to form 1 mol of MgO at 25 ℃ (298 K):

$$\Delta H = \Delta_{298}^{\Theta} = (-285.83)+(-601.24)+(-924.66) \text{ = 37.593kJ/mol = 939.825 kJ/kg,}$$

Q_1 = 939.825 kJ/kg × 1.121 kg × 35.05% = 369.27 kJ

The heat required to heat MgO:

$$\int_{298}^{1273}(48.99+3.14\times10^{-3}\cdot T-11.72\times10^{5}/T^2)\mathrm{d}T = \text{ 47.2 kJ/mol} = \text{1180 kJ/kg}$$

Q_2 = 1180 kJ/kg × (1.121 kg × 35.05% × 40/58 + 1.121 × 52.25%) = 1010.90 kJ

The heat required to heat H_2O:

$$\int_{298}^{1273}(-203.60+1523.29\cdot T-3193.41\cdot T^2+2474.45\cdot T^3+3.86/T^2)\mathrm{d}T = \text{ 78.7 kJ/mol} = \text{23900 kJ/kg}$$

Q_3 = 23900 kJ/kg × 1.121 kg × 35.05% × 10/58 = 2914.31 kJ

The theoretical power consumption:

$$Q_{total} = Q_1 + Q_2 + Q_3 = 369.27 \text{ kJ} + 1010.9 \text{ kJ} + 2914.31 \text{ kJ} = 4294.48 \text{ kJ} = 1.193 \text{ kWh}$$

The mechanical energy consumption of ball milling 1 kg of clinker was estimated to be 0.016 kWh. Therefore, the total energy consumption for the production of 1 kg of DB-MgO from magnesium residue was 1.209kWh, as shown in Table S4.

Table S4 Inventory of theoretical production processes for DB-MgO (1 kg)

Inputs/Emissions		Amount
Raw materials	Magnesium residue (kg)	1.121
Energy	Electricity (kWh)	1.209
Emissions	Water vapor (kg)	0.121

S5. Inventory of production of KDP by the neutralization method

The raw material consumption of the neutralization method was based on theoretical numerical calculations, and the energy consumption data were from the Refs. [4, 5], as shown in Table S4.

Table S5 Inventory of production of 1 kg of KDP [4, 5]

Inputs/Emissions		Amount
Raw materials	Potassium hydroxide (KOH) (g)	412.00
	Phosphoric acid solution (85% H_3PO_4) (g)	847.06
	Distilled water (g)	1554.27
Energy	Hard coal (kg)	1.20
	Electricity (kWh)	0.20

S6. Sensitivity analysis results

The sensitivity analysis results for various magnesium-based cement are summarized in Tables S6-S15.

Table S6 Sensitivity analysis results of BMSC-D

Impact category	Unit	Mean	Standard deviation	Coefficient of variation	Minimum	Maximum	Median
ODP	kg CFC-11-eq.	1.91325E-08	1.29044E-11	0.07%	1.911E-08	1.91552E-08	1.91321E-08
GWP100a	kg CO_2-eq.	1.738820268	0.085399398	4.91%	1.538437066	1.929117664	1.73468638
FETP	kg 1,4-DB-eq.	0.393747265	0.004849969	1.23%	0.385260865	0.402247172	0.393595834
FDP	MJ	8.592959002	0.356235476	4.15%	7.969623673	9.217286478	8.581836236
AP	kg SO_2-eq.	0.006641533	0.000301578	4.54%	0.006018289	0.007244008	0.006646187
HTP	kg 1,4-DB-eq.	0.616051374	0.003565572	0.58%	0.609842174	0.622394548	0.615967962
TETP	kg 1,4-DB-eq.	0.001631947	6.65499E-06	0.41%	0.001620302	0.00164361	0.001631739
POCP	kg C_2H_4-eq.	0.000466982	2.31735E-05	4.96%	0.000413473	0.00051982	0.000467121
MDP	kg Sb-eq.	4.79705E-06	3.2342E-09	0.07%	4.79139E-06	4.80272E-06	4.79695E-06
METP	kg 1,4-DB-eq.	843.6411022	12.38445367	1.47%	821.9709766	865.3457195	843.2544215
EP	kg PO_4-eq.	0.001051864	2.70431E-05	2.57%	0.001004463	0.001099188	0.001050976

ODP: Ozone depletion potential; GWP100a: Global warming potential; FETP: Freshwater ecotoxicity; FDP: Fossil fuel depletion potential; AP: Acidification potential; HTP: Human toxicity potential; TETP: Terrestrial ecotoxicity potential; POCP: Photochemical ozone creation potential; MDP: Metal/mineral resource depletion potential; METP: Marine ecotoxicity potential; EP: Eutrophication potential.

Table S7 Sensitivity analysis results of BMSC-B

Impact category	Unit	Mean	Standard deviation	Coefficient of variation	Minimum	Maximum	Median
ODP	kg CFC-11-eq.	2.19744E-08	2.3429E-11	0.11%	2.19315E-08	2.20168E-08	2.19756E-08
GWP100a	kg CO_2-eq.	0.864658205	0.022477647	2.60%	0.811076581	0.914103059	0.865834692
FETP	kg 1,4-DB-eq.	0.439025815	0.003774268	0.86%	0.431481692	0.446601728	0.439146488
FDP	MJ	7.475003048	0.159369018	2.13%	7.125833004	7.827143345	7.477465029
AP	kg SO_2-eq.	0.004284693	8.54308E-05	1.99%	0.004071507	0.004492643	0.004284978
HTP	kg 1,4-DB-eq.	0.724750058	0.005100106	0.70%	0.715240907	0.734197751	0.724935839
TETP	kg 1,4-DB-eq.	0.001973705	1.51379E-05	0.77%	0.001946302	0.002000606	0.00197424
POCP	kg C_2H_4-eq.	0.000205165	5.03667E-06	2.45%	0.000193183	0.000218122	0.000205023
MDP	kg Sb-eq.	5.48336E-06	3.06314E-09	0.06%	5.47739E-06	5.48934E-06	5.48349E-06
METP	kg 1,4-DB-eq.	1106.34834	24.75821902	2.24%	1061.247237	1150.780172	1107.570527
EP	kg PO_4-eq.	0.001074814	1.63646E-05	1.52%	0.001040829	0.001109074	0.001075554

Table S8 Sensitivity analysis results of BMSC-FA

Impact category	Unit	Mean	Standard deviation	Coefficient of variation	Minimum	Maximum	Median
ODP	kg CFC-11-eq.	1.58691E-08	1.11159E-11	0.07%	1.58502E-08	1.58876E-08	1.58686E-08
GWP100a	kg CO_2-eq.	1.443097295	0.070586347	4.89%	1.281602817	1.60895102	1.446289481
FETP	kg 1,4-DB-eq.	0.32678946	0.004177773	1.28%	0.319662537	0.333733402	0.326590383
FDP	MJ	7.139080783	0.306862003	4.30%	6.615600501	7.649120838	7.124458315
AP	kg SO_2-eq.	0.005503075	0.000253524	4.61%	0.005001132	0.006027998	0.005496678
HTP	kg 1,4-DB-eq.	0.511202766	0.003070321	0.60%	0.505909232	0.516348216	0.511004878
TETP	kg 1,4-DB-eq.	0.001354166	5.73263E-06	0.42%	0.001344386	0.001363694	0.001353892
POCP	kg C_2H_4-eq.	0.000387629	1.99523E-05	5.15%	0.000343662	0.000433092	0.000387799
MDP	kg Sb-eq.	3.97885E-06	2.78594E-09	0.07%	3.97409E-06	3.98348E-06	3.97871E-06
METP	kg 1,4-DB-eq.	700.2524601	10.66799496	1.52%	682.0537749	717.9838986	699.744113
EP	kg PO_4-eq.	0.000873321	2.32926E-05	2.67%	0.000833506	0.000912109	0.000872194

Table S9 Sensitivity analysis results of BMSC-S

Impact category	Unit	Mean	Standard deviation	Coefficient of variation	Minimum	Maximum	Median
ODP	kg CFC-11-eq.	1.58685E-08	1.06955E-11	0.07%	1.58502E-08	1.58877E-08	1.58683E-08
GWP100a	kg CO_2-eq.	1.445157815	0.07027862	4.86%	1.276513662	1.60530884	1.446485676
FETP	kg 1,4-DB-eq.	0.32653864	0.004019775	1.23%	0.319657535	0.333752859	0.326479838
FDP	MJ	7.120067982	0.295256695	4.15%	6.614666044	7.649963298	7.115753549
AP	kg SO_2-eq.	0.005501462	0.00024594	4.47%	0.005004808	0.006003259	0.005493571
HTP	kg 1,4-DB-eq.	0.511024196	0.002953025	0.58%	0.505936017	0.516376285	0.510962408
TETP	kg 1,4-DB-eq.	0.00135383	5.51583E-06	0.41%	0.001344388	0.001363729	0.001353749
POCP	kg C_2H_4-eq.	0.000386633	1.85119E-05	4.79%	0.000342813	0.00043069	0.00038687
MDP	kg Sb-eq.	3.97875E-06	2.6806E-09	0.07%	3.97416E-06	3.98356E-06	3.97871E-06
METP	kg 1,4-DB-eq.	699.6154825	10.26454576	1.47%	682.0443623	718.0370602	699.4653026
EP	kg PO_4-eq.	0.000871901	2.24126E-05	2.57%	0.00083351	0.000912175	0.000871543

Table S10 Sensitivity analysis results of CRMC-B

Impact category	Unit	Mean	Standard deviation	Coefficient of variation	Minimum	Maximum	Median
ODP	kg CFC-11-eq.	5.26251E-10	4.61074E-11	8.76%	4.49646E-10	6.08101E-10	5.22509E-10
GWP100a	kg CO_2-eq.	0.366033845	0.032070027	8.76%	0.312751274	0.422964646	0.363431038
FETP	kg 1,4-DB-eq.	0.082809066	0.00725531	8.76%	0.070754771	0.095688712	0.082220224
FDP	MJ	3.271551366	0.286636723	8.76%	2.795320355	3.780389668	3.248287895
AP	kg SO_2-eq.	0.001474421	0.000129181	8.76%	0.001259794	0.001703744	0.001463937
HTP	kg 1,4-DB-eq.	0.114204809	0.010006046	8.76%	0.097580319	0.131967568	0.113392717
TETP	kg 1,4-DB-eq.	0.000340609	2.98424E-05	8.76%	0.000291027	0.000393585	0.000338187
POCP	kg C_2H_4-eq.	5.46718E-05	4.79007E-06	8.76%	4.67134E-05	6.31752E-05	5.42831E-05
MDP	kg Sb-eq.	6.78586E-08	5.94543E-09	8.76%	5.79806E-08	7.84129E-08	6.73761E-08
METP	kg 1,4-DB-eq.	556.57713	48.76446273	8.76%	475.5576808	643.1439388	552.6194002
EP	kg PO_4-eq.	0.000351832	3.08257E-05	8.76%	0.000300617	0.000406554	0.00034933

Table S11 Sensitivity analysis results of CRMC-L

Impact category	Unit	Mean	Standard deviation	Coefficient of variation	Minimum	Maximum	Median
ODP	kg CFC-11-eq.	2.59394E-10	1.9125E-11	7.37%	2.27212E-10	2.93402E-10	2.59194E-10
GWP100a	kg CO_2-eq.	2.000051827	0.122353035	6.12%	1.714010059	2.293522917	1.996201581
FETP	kg 1,4-DB-eq.	0.084467413	0.007187917	8.51%	0.072372428	0.097248919	0.084392496
FDP	MJ	6.168893997	0.527960345	8.56%	5.280504187	7.107709533	6.163391299
AP	kg SO_2-eq.	0.006218048	0.000457272	7.35%	0.005315284	0.007090432	0.006227122
HTP	kg 1,4-DB-eq.	0.064524337	0.005277876	8.18%	0.055571086	0.074026195	0.064503917
TETP	kg 1,4-DB-eq.	0.000121072	9.86306E-06	8.15%	0.000104475	0.00013861	0.000120969
POCP	kg C_2H_4-eq.	0.000544491	3.46212E-05	6.36%	0.000468847	0.000623676	0.000542167
MDP	kg Sb-eq.	5.69155E-08	4.79326E-09	8.42%	4.885E-08	6.54388E-08	5.68655E-08
METP	kg 1,4-DB-eq.	222.7729345	18.35443371	8.24%	191.8882439	255.4106651	222.5816343
EP	kg PO_4-eq.	0.000470544	4.00721E-05	8.52%	0.000402941	0.000541963	0.000470108

Table S12 Sensitivity analysis results of MKPC-D

Impact category	Unit	Mean	Standard deviation	Coefficient of variation	Minimum	Maximum	Median
ODP	kg CFC-11-eq.	1.65816E-08	9.93088E-10	5.99%	1.45261E-08	1.88509E-08	1.65344E-08
GWP100a	kg CO_2-eq.	3.970806345	0.165430392	4.17%	3.535232871	4.388047861	3.979351647
FETP	kg 1,4-DB-eq.	2.050196105	0.136205808	6.64%	1.794394106	2.347705018	2.044141694
FDP	MJ	26.49456713	1.152792747	4.35%	23.29293585	29.82375617	26.51305268
AP	kg SO_2-eq.	0.01745201	0.000877461	5.03%	0.015525382	0.019720182	0.017406168
HTP	kg 1,4-DB-eq.	2.386609983	0.158533956	6.64%	2.081388832	2.731547426	2.380225176
TETP	kg 1,4-DB-eq.	0.003331857	0.000208732	6.26%	0.002921944	0.003799654	0.003322719
POCP	kg C_2H_4-eq.	0.001499458	6.77169E-05	4.52%	0.001330862	0.001697655	0.001498746
MDP	kg Sb-eq.	7.75295E-05	2.48673E-06	3.21%	7.29359E-05	8.25201E-05	7.74132E-05
METP	kg 1,4-DB-eq.	4463.142057	279.6484203	6.27%	3928.826049	5089.463863	4450.454723
EP	kg PO_4-eq.	0.004865782	0.000266014	5.47%	0.004291908	0.005543782	0.004859361

Table S13 Sensitivity analysis results of MKPC-B

Impact category	Unit	Mean	Standard deviation	Coefficient of variation	Minimum	Maximum	Median
ODP	kg CFC-11-eq.	1.66177E-08	9.37971E-10	5.64%	1.45038E-08	1.87209E-08	1.66286E-08
GWP100a	kg CO_2-eq.	2.928627228	0.138535343	4.73%	2.570975323	3.278099233	2.925476452
FETP	kg 1,4-DB-eq.	2.027692408	0.128444095	6.33%	1.751659747	2.296701537	2.027313165
FDP	MJ	25.03444862	1.064728052	4.25%	22.08768508	27.91237163	24.99002223
AP	kg SO_2-eq.	0.015300262	0.000804252	5.26%	0.013307373	0.017202032	0.015310727
HTP	kg 1,4-DB-eq.	2.425196004	0.150272951	6.20%	2.09652157	2.749433585	2.42406999
TETP	kg 1,4-DB-eq.	0.00367175	0.000203758	5.55%	0.00318724	0.004143971	0.003669761
POCP	kg C_2H_4-eq.	0.001078733	5.48944E-05	5.09%	0.000938623	0.001222007	0.001078194
MDP	kg Sb-eq.	7.73678E-05	2.34328E-06	3.03%	7.28771E-05	8.19392E-05	7.73597E-05
METP	kg 1,4-DB-eq.	5032.523481	277.1767204	5.51%	4367.514894	5671.598105	5030.242049
EP	kg PO_4-eq.	0.004817334	0.000250987	5.21%	0.004191385	0.005426859	0.004820614

Table S14 Sensitivity analysis results of MKPC-B-FA

Impact category	Unit	Mean	Standard deviation	Coefficient of variation	Minimum	Maximum	Median
ODP	kg CFC-11-eq.	1.00877E-08	6.29723E-10	6.24%	8.71316E-09	1.14588E-08	1.00789E-08
GWP100a	kg CO_2-eq.	1.886309069	0.092010405	4.88%	1.664384892	2.120428669	1.886508926
FETP	kg 1,4-DB-eq.	1.295407976	0.085669183	6.61%	1.123911866	1.46243131	1.295529343
FDP	MJ	15.90629143	0.718201348	4.52%	14.05013281	17.84833906	15.88887579
AP	kg SO_2-eq.	0.009872524	0.000538658	5.46%	0.008651558	0.01110158	0.00986862
HTP	kg 1,4-DB-eq.	1.551611083	0.100226978	6.46%	1.343476682	1.755349016	1.550526167
TETP	kg 1,4-DB-eq.	0.002370929	0.000135912	5.73%	0.002063011	0.00266597	0.002369243
POCP	kg C_2H_4-eq.	0.000690457	3.68778E-05	5.34%	0.000604282	0.000780758	0.000689614
MDP	kg Sb-eq.	2.0144E-05	1.56049E-06	7.75%	1.71773E-05	2.30521E-05	2.01119E-05
METP	kg 1,4-DB-eq.	3285.084388	184.8281935	5.63%	2871.580398	3696.577044	3283.829031
EP	kg PO_4-eq.	0.003081704	0.000168865	5.48%	0.002696685	0.003459408	0.003080934

Table S15 Sensitivity analysis results of MKPC-B-S

Impact category	Unit	Mean	Standard deviation	Coefficient of variation	Minimum	Maximum	Median
ODP	kg CFC-11-eq.	1.22659E-08	7.56945E-10	6.17%	1.05996E-08	1.39648E-08	1.22507E-08
GWP100a	kg CO_2-eq.	2.30267645	0.111302735	4.83%	2.029149853	2.593950917	2.302093501
FETP	kg 1,4-DB-eq.	1.574180417	0.102998688	6.54%	1.372344243	1.781432841	1.577633515
FDP	MJ	19.416499	0.873322123	4.50%	17.10807506	21.78666818	19.40132598
AP	kg SO_2-eq.	0.012015165	0.000651497	5.42%	0.010597185	0.013496144	0.012032193
HTP	kg 1,4-DB-eq.	1.885986904	0.120798062	6.41%	1.637298887	2.142882283	1.885876103
TETP	kg 1,4-DB-eq.	0.002884794	0.000165091	5.72%	0.002530597	0.003259158	0.002886222
POCP	kg C_2H_4-eq.	0.000841189	4.31732E-05	5.13%	0.000737344	0.00094525	0.000839634
MDP	kg Sb-eq.	2.44502E-05	1.87673E-06	7.68%	2.08696E-05	2.81207E-05	2.44383E-05
METP	kg 1,4-DB-eq.	3997.850638	224.943712	5.63%	3511.969069	4502.347171	4001.958094
EP	kg PO_4-eq.	0.003749844	0.000202561	5.40%	0.00330289	0.004213808	0.003748652

References

(1) Ruan, S., Unluer, C., 2016. Comparative life cycle assessment of reactive MgO and Portland cement production. Journal of Cleaner Production 137, 258-273.

(2) Xu, T. (2020). Numerical Calculation of Gas-Solid HeatTransfer Process of Magnesite Reburning Shaft Kiln (Master's thesis). Northeastern University, Shenyang, China. (in Chinese)

(3) Piccinno, F., et al., 2016. From laboratory to industrial scale: a scale-up framework for chemical processes in life cycle assessment studies. Journal of Cleaner Production 135(nov.1), 1085-1097.

(4) Lei, W., 2004. The production of potassium dihydrogen phosphate from wet -process phosphoric acid. Inorganic Chemicals Industry 36(02), 44-45. (in Chinese).

(5) Ping, C. (2016). Study on new technology of preparation of potassium dihydrogen phosphate with wet process phosphoric acid (Master's thesis). Wuhan Institute of Technology, Wuhan, China. (in Chinese)
